# Recent advances in understanding dominant spinocerebellar ataxias from clinical and genetic points of view

**DOI:** 10.12688/f1000research.15788.1

**Published:** 2018-11-12

**Authors:** Giulia Coarelli, Alexis Brice, Alexandra Durr

**Affiliations:** 1 Assistance Publique-Hôpitaux de Paris (AP-HP), Department of Neurology, Avicenne Hospital, Paris 13 University, Bobigny, 93000, France; 2 Institut du Cerveau et de la Moelle épinière, ICM, Inserm U 1127, CNRS UMR 7225, Sorbonne University, Paris, 75013, France; 3 Assistance Publique-Hôpitaux de Paris (AP-HP), Genetic department, Pitié-Salpêtrière University Hospital, Paris, 75013, France

**Keywords:** spinocerebellar ataxias, biomarkers, antisense oligonucleotides, clinical trials, neuroimaging

## Abstract

**Abstract**

Spinocerebellar ataxias (SCAs) are rare types of cerebellar ataxia with a dominant mode of inheritance. To date, 47 SCA subtypes have been identified, and the number of genes implicated in SCAs is continually increasing. Polyglutamine (polyQ) expansion diseases

( *ATXN1*/SCA1, *ATXN2*/SCA2, *ATXN3*/SCA3, *CACNA1A*/SCA6, *ATXN7*/SCA7, *TBP*/SCA17, and *ATN1*/DRPLA) are the most common group of SCAs. No preventive or curative treatments are currently available, but various therapeutic approaches, including RNA-targeting treatments, such as antisense oligonucleotides (ASOs), are being developed. Clinical trials of ASOs in SCA patients are already planned. There is, therefore, a need to identify valid outcome measures for such studies. In this review, we describe recent advances towards identifying appropriate biomarkers, which are essential for monitoring disease progression and treatment efficacy. Neuroimaging biomarkers are the most powerful markers identified to date, making it possible to reduce sample sizes for clinical trials. Changes on brain MRI are already evident at the premanifest stage in SCA1 and SCA2 carriers and are correlated with CAG repeat size. Other potential biomarkers have also been developed, based on neurological examination, oculomotor study, cognitive assessment, and blood and cerebrospinal fluid analysis. Longitudinal studies based on multimodal approaches are required to establish the relationships between parameters and to validate the biomarkers identified.

## Introduction

Spinocerebellar ataxias (SCAs) are a group of neurodegenerative diseases displaying autosomal dominant inheritance. To date, 47 SCA subtypes have been described, and 35 causal genes have been identified ^1^. SCAs are considered a rare group of cerebellar ataxias, with a mean prevalence of 2.7/100,000 ^2^. Their most frequent forms are polyglutamine (polyQ) expansion diseases ( *ATXN1*/SCA1, *ATXN2*/SCA2, *ATXN3*/SCA3, *CACNA1A*/SCA6, *ATXN7*/SCA7, *TBP*/SCA17, and *ATN1*/DRPLA) ^3^. These diseases manifest above a threshold number of CAG repeats, which is different for each gene. The same mutational mechanism is found in Huntington disease ^4^ and in spinal bulbar muscular atrophy ^5^. Disease onset generally occurs between the ages of 20 and 40 years, and age at onset and CAG repeat expansion size are inversely correlated ^6, 7^. Age at onset also displays variability due to interactions between polyQ genes, i.e. between the expanded allele and the alleles of normal repeat size in the other genes ^8, 9^. The instability of expanded alleles results in genetic anticipation in successive generations ^3^. Unsteady gait and clumsiness are usually the first clinical symptoms at onset, followed by a progressive loss of the ability to walk. Cerebellar syndrome is often associated with extracerebellar signs (pyramidal syndrome, extrapyramidal syndrome, retinal degeneration, dementia, seizures, etc.) ^3^. There is currently no preventive or curative treatment, but different therapeutic approaches are being tested, including the use of disease-modifying compounds and therapeutic approaches, which are described below. However, in SCAs, the lack of sensitive biomarkers and the small number of patients to be included in clinical trials are a challenge for the statistical sample size estimation. Outcome measures providing information about treatment efficacy are vital, particularly for these genetic diseases that can be treated before the onset of symptoms. This review provides an overview of findings for biomarkers, recent results of clinical trials in SCA patients, and future perspectives for treatment.

## Natural history of spinocerebellar ataxias (SCA1, 2, 3, and 6)

The natural history of SCA1, 2, 3, and 6 was established by the longitudinal European cohort study EUROSCA, which included 462 patients with at least one year of follow-up and a median of 49 months of observation ^10^. This study used the Scale for the Assessment and Rating of Ataxia (SARA), an accurate measurement of cerebellar dysfunction ^11, 12^ in SCA1, 2, 3, and 6 patients and premanifest individuals ^13^. The most severe disease turned out to be SCA1, with an annual progression of 2.11 on the 40-point SARA, followed by SCA3 (1.56 points), SCA2 (1.49 points), and SCA6 (0.8 points) ^10^. SCA1 has also been reported to have the most severe prognosis for progression in other studies, such as one in Europe ^14^ and another conducted by the North American consortium (the Clinical Research Consortium for Spinocerebellar Ataxias) ^15^. This may be because of motoneuron involvement, which, particularly in this form, leads to bulbar dysfunction and, thus, to respiratory and swallowing failure ^16^. These results are consistent with 10-year survival, which is lowest for SCA1 patients, intermediate for SCA2 and SCA3, and highest for SCA6 patients ^17^. A high SARA score has also been identified as one of the strongest risk factors for death in all subtypes. Other associated risk factors include dysphagia for SCA1, longer CAG length in pathologic allele for SCA2, and dystonia and interaction between age and CAG length in SCA3 ^17^. The Composite Cerebellar Functional Severity (CCFS) score is another quantitative score for assessing cerebellar ataxia that has been validated in adults and children ^18, 19^. This score is calculated from scores for two tasks for the dominant hand—the nine-hole pegboard test and the click test—and is age dependent and available from an open source ( https://icm-institute.org/en/tutorial-for-making-ccfs-board/). In a study on Friedreich ataxia and SCA1, 2, 3, and 7 patients, SARA and CCFS scores were higher in SCAs than in Friedreich ataxia after adjustment for disease duration, revealing a slower progression in Friedreich ataxia than in SCAs. Considering each SCA subtype separately, SCA2 and SCA1 patients had higher CCFS scores than did patients with other subtypes, despite an absence of difference in SARA scores ^20^, probably because of the more accentuate cerebellar syndrome.

**Figure 1.**
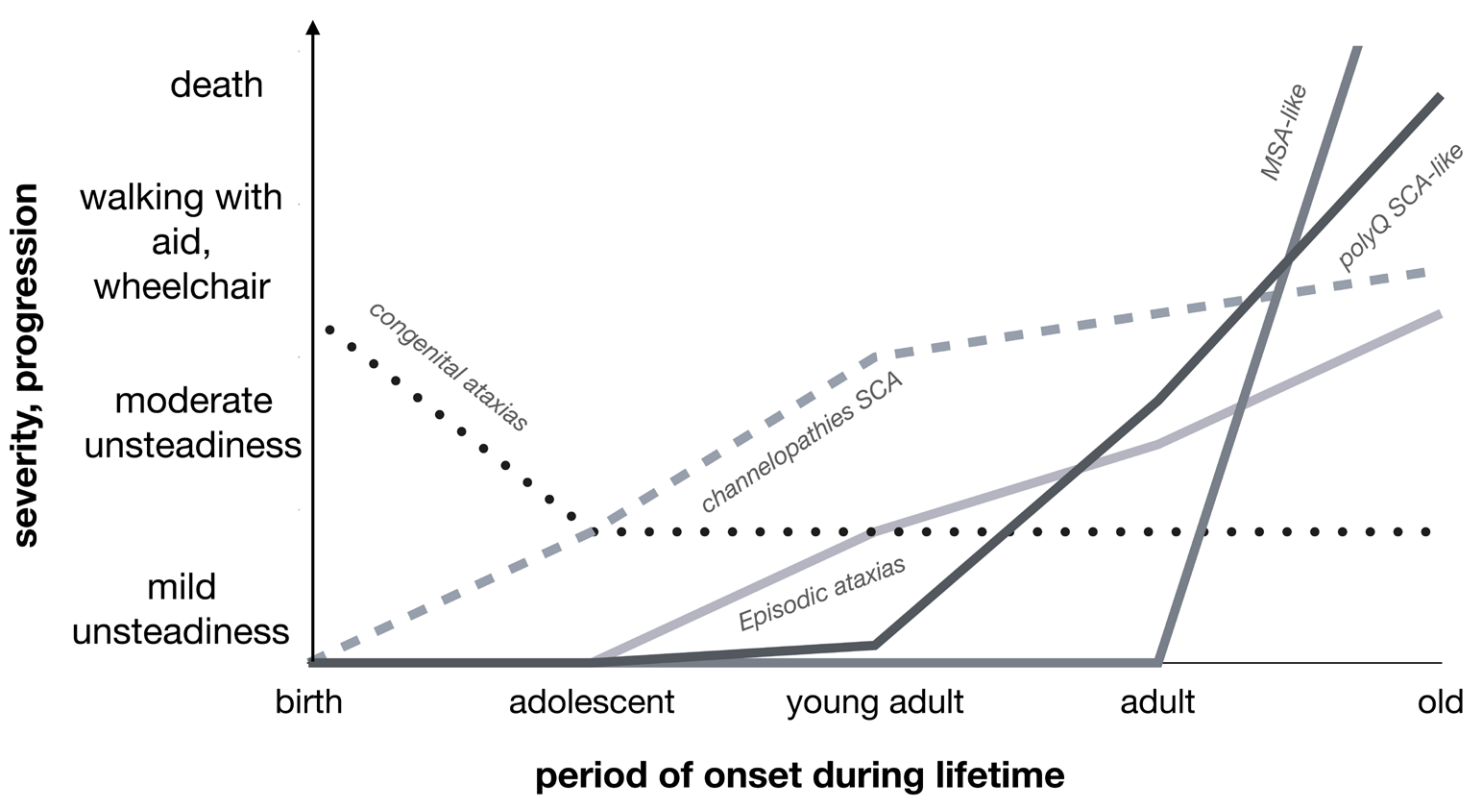
Progression and severity profiles in cerebellar ataxias by underlying etiology Multisystem atrophy (MSA) is a rapidly progressing non-monogenic alpha-synucleinopathy. It is the most severe of these diseases, followed by polyglutamine (polyQ) spinocerebellar ataxias (SCAs), such as *ATXN1*/SCA1, *ATXN2*/SCA2, *ATXN3*/SCA3, *CACNA1A*/SCA6, *ATXN7*/SCA7, *TBP*/SCA17, and *ATN1*/DRPLA. Very different profiles are present in the forms because of missense mutations in channel genes ( *CACNA1A*, *KCND3*, *KCNC3*, and *KCNA1)*.

Based on SARA score progression, the EUROSCA study identified, for each genotype, the sample sizes required to detect a 50% decrease in SARA score progression in a two-arm clinical trial lasting one year: 142 patients for SCA1, 172 for SCA2, 202 for SCA3, and 602 for SCA6 ^10^. These are large numbers of patients for such rare diseases, making it necessary to carry out clinical trials at an international level. Additional biomarkers are required to overcome this problem. Furthermore, these dominantly inherited cerebellar ataxias have very different progression profiles, as shown in Figure 1. Given the progressive nature of the disease and the sample sizes required, biomarkers are very important and essential.

## Biomarkers

### Neuroimaging biomarkers

The most powerful biomarkers identified to date are derived from neuroimaging examinations. Specific patterns of brain atrophy have been described *in vivo* in polyQ SCAs ^21, 22^ and confirmed in post-mortem studies ^23, 24^. Changes in brain MRI findings are already evident at the premanifest stage in SCA1 and SCA2 carriers in the form of losses of gray matter from the cerebellum and brainstem. In SCA2 carriers only, an additional decrease in brainstem volume relative to non-carriers has also been reported ^13^. A recent case-control study on a Cuban-German cohort of premanifest and manifest SCA2 carriers reported remarkable decreases in cerebellum and pontine volume and in the anteroposterior diameter of the pontine brainstem. A negative correlation was found between CAG repeat size and brainstem and cerebellum volume in premanifest individuals ^25^. Cerebellar and pons atrophy was more pronounced in manifest patients ^25^, representing a potential outcome measure. By contrast, midbrain and medulla volumes did not differ significantly between the preclinical and clinical stages ^25^. In SCA2, white matter alterations in the parietal lobe and anterior corona radiata (detected by fractional anisotropy), and in the cerebellum and middle cerebellar peduncle (detected by mean diffusivity), were found to be correlated with SARA scores, leading the authors of the report concerned to suggest that connections between the motor and sensory integration areas might be impaired ^26^. However, with the exception of rare follow-up studies ^21, 27^, only cross-sectional brain MRI studies have been performed to date. Longitudinal studies will be required to establish the rate of progression of cerebellar and brainstem atrophy.

Cervical spinal cord atrophy has been proposed as a possible biomarker for SCA1, as it is correlated with SARA score, CAG repeat length, and disease duration ^28^. Indeed, the clinical presentation of this subtype can include pyramidal signs and it may, at early stages, mimic spastic paraplegia ^29^. Cervical spinal cord atrophy has also been identified as a potential biomarker for SCA3 ^30^.

Neurochemical abnormalities can be detected by MRI spectroscopy (MRS), as demonstrated by the group headed by Gulin Oz ^31^. Decreases in N-acetylaspartate and glutamate levels reflect a loss of neurons, whereas increases in myoinositol serve as a marker of gliosis. N-acetylaspartate and N-acetylaspartylglutamate levels are significantly lower in the vermis and pons of SCA1, 2, 3, and 7 patients than in controls. Myoinositol levels are higher in SCA1, 2, and 3 patients than in controls. This neurochemical profile is particularly evident in SCA2 and SCA3 patients and is correlated with SARA score ^32^. Interestingly, multicenter acquisition of this technique has been validated ^32^.

Such alterations have been demonstrated by ultra-high-field MRS, even at the premanifest stage, with SCA2 patients showing the most compromised profile, followed by SCA1, SCA3, and SCA6 variant carriers ^31^.

Another recent neuroimaging technique applied to SCAs is functional MRI (fMRI) at rest ^33 ^or during a task ^34^. This technique can be used to characterize circuitry reorganization, making it possible to identify specific patterns of cerebellar activation ^35, 36^. Using this approach in the early phase of SCA3 disease, Duarte *et al*. discovered a reorganization of the motor network that could potentially serve as a biomarker ^37^. However, more detailed analyses of fMRI outcomes are required for future application.

Neuroimaging biomarkers are more powerful than clinical scores for the detection of disease progression. In a cohort of SCA1, 2, 3, and 7 patients, greater longitudinal effect size was detected for brain volumetry (>1.2) than SARA and CCFS scores (<0.8) ^27^. In conclusion, measures of pons and cerebellum atrophy seem to be the most promising biomarkers.

### Oculomotor biomarkers

Oculomotor involvement is present at an early phase of SCA and is detectable even at premanifest stages as a higher rate of gaze-evoked nystagmus or other alterations, such as a square-wave jerk during central fixation, impaired vertical smooth pursuit, slow saccade, and a higher antisaccade error rate in SCA3 carriers than controls ^13, 38, 39^. These alterations have been studied in detail in SCA2 patients, all of whom present specific oculomotor abnormalities (slow horizontal saccades up to saccade paresis) ^40^. Preclinical SCA2 carriers present a reduced saccade velocity and antisaccade task errors ^41^.

Moreover, in manifest and premanifest SCA2 carriers, both the progression of oculomotor impairment and the slowing of horizontal saccade velocity are correlated, respectively, with CAG repeat size ^42^ and pontine atrophy ^25^. The annual decrease in saccade velocity and saccade accuracy and an increase in saccade latency may soon be used as biomarkers. This correlation between saccade measurements and other parameters, such as brain MRI and/or cognitive assessment, may make it possible to reduce sample size in future trials ^42^.

## Biological biomarkers

No biological biomarkers for SCAs have yet been identified, a situation contrasting with other neurodegenerative diseases, such as Alzheimer’s disease and frontotemporal dementia. There is, therefore, a need to search for blood or cerebrospinal fluid (CSF) biomarkers of these diseases. In addition to guiding diagnostic process, biomarkers may be of use in clinical management if linked to disease progression.

In this respect, several recent studies in patients with SCA3, the most frequent SCA subtype, have yielded promising results. *SIRT1* encodes sirtuin-1, an NAD ^+^-dependent deacetylase involved in several cellular functions, including chromatin modulation, the cell cycle, apoptosis, and autophagy regulation in response to DNA damage. SIRT1 mRNA levels are lower in SCA3 mice than in wild-type mice and are also low in the fibroblasts of SCA3 patients ^43^. In SCA3 mice, the rescue of SIRT1 by caloric restriction or the administration of resveratrol induces motor improvement and neuropathological changes, such as a decrease in neuroinflammation and reactive gliosis with an activation of autophagy ^43^. *SIRT1* overexpression has been shown to activate autophagy and thus to induce higher levels of mutant protein clearance. For these reasons, resveratrol may be a useful neuroprotective drug, as shown in some assays in *Drosophila* models of SCA3 ^44^ and in the rat 3-acetylpyridine-induced cerebellar ataxia model ^45^. Other studies have also demonstrated an imbalance between autophagy and apoptosis in SCA3. For example, an increase has been reported in the levels of *BECN1*, encoding the proautophagic Beclin 1 protein ^46^, and BCL2/BAX ratio has been shown to be lower in pre-ataxic SCA3 carriers than in controls ^47^, enhancing apoptotic processes.

Oxidative stress has been implicated in several neurodegenerative disorders, and SCA3 patients have been shown to produce abnormally large amounts of reactive oxygen species ^48^. This phenomenon results from a decrease in antioxidant capacity: both superoxide dismutase and glutathione peroxidase (GPx) activities are lower in symptomatic than in pre-symptomatic carriers ^49^. Moreover, the observed correlation of the decrease in GPx levels with disease severity suggests that GPx may be a reliable biomarker ^49^.

Cytokines have also been investigated as possible markers of SCA3. Indeed, enhanced inflammation has been linked to stronger staining for IL1B and IL6 and higher levels of activated microglia and reactive astrocytes in the brains of SCA3 patients ^50^. In a study on Brazilian SCA3 patients, no difference in cytokine levels was detected between 79 carriers and 43 controls. On the contrary, higher eotaxin levels were observed in asymptomatic carriers than in symptomatic carriers. It has been suggested that the levels of eotaxin released by astrocytes are inversely correlated with disease progression ^51^. In another cohort of SCA3 patients recruited in the Azores, lower IL6 mRNA levels, due to the presence of the IL6*C allele, were associated with an earlier age at onset than the presence of the IL6*G allele ^52^. In this study, the earlier age at onset (by about 10 years on average) resulted from the presence of the APOE*e2 allele in IL6*C carriers, probably because of additional decreases in the levels of other cytokines, such as IL1B and TNF, due to the presence of the APOE*e2 allele ^53^.

In a recent study, serum neurofilament light chain (Nfl) was identified as a powerful potential serum biomarker in polyQ SCAs ^54^. Neurofilaments are components of the neuron cytoskeleton, and their levels in the blood reflect damage to the axons of long fiber tracts ^55^. In Huntington disease, which is also a polyQ disease, a correlation between serum Nfl levels and disease severity (UHDRS, cognitive decline, brain atrophy) has been reported, even after adjustment for age and CAG repeat size ^56^. Nfl dosage is also useful for predicting disease onset in pre-symptomatic carriers and the progression of Huntington disease ^56^. In a study by Wilke *et al*., Nfl levels were higher in SCAs patients than in controls, particularly for SCA1 and SCA3 ^54^. The CSF has been only briefly explored in SCAs. CSF Nfl levels are a potential diagnostic and prognostic biomarker for SCAs, as in amyotrophic lateral sclerosis ^57^. A study in SCA1, SCA2, and SCA6 patients evaluated α-synuclein, DJ-1, and glial fibrillary acidic protein levels in CSF. The levels of all of these proteins were higher in CSF from patients than in control CSF, but this difference was significant only for tau, the levels of which were significantly higher in SCA2 patients. No correlations were found for CAG repeat size, disease severity, and disease duration ^58^. It will be very interesting to determine polyQ protein levels in the CSF of SCA patients, as has been done already for Huntington disease.

### Cognition in spinocerebellar ataxias

Cognitive impairment, of various degrees of severity, can occur in polyQ SCAs. It involves mostly the executive functions and verbal memory, as shown in one of the first cognitive studies to compare the profiles of SCA1, 2, and 3 patients ^59^. Cognitive decline was more prominent and rapid in SCA1 than in the other genotypes ^59– 61^ and was associated with an increase in the incidence of depression ^61^. In another study, SCA2 patients were found to have more visuospatial and visuoperceptive deficits than SCA1 patients ^62^. In a recent study focusing on cognitive dysfunction in SCA6 patients, mild impairments of executive functions, mental flexibility, and visuospatial skills were found to be correlated with decreased resting-state connectivity in the frontoparietal network ^63^. These data support a role for the cerebellum in cognitive processes, given that the cerebellum is the principal cerebral structure affected and neuronal loss is less severe in SCA6 than in other polyQ diseases ^23^. The scales most widely used to assess dementia, such as the Mini-Mental State Examination or Montreal Cognitive Assessment, are not appropriate for the detection of cognitive impairment in SCA patients, who present a cerebellar cognitive affective/Schmahmann’s syndrome (CCAS) ^64^. This syndrome includes executive dysfunctions, spatial cognition difficulties, language deficits, and personality changes. A CCAS scale has recently been validated in a large cohort of cerebellar patients with acquired, genetic, or idiopathic cause and shown to have a sensitivity of 95% and a selectivity of 78% for CCAS diagnosis ^64^. This scale, which is easy and fast to administer, can be used for cognitive evaluation, which should be performed in longitudinal studies of patients.

## Genetic advances in spinocerebellar ataxias

The number of genes implicated in SCAs has steadily increased over time. The last causal gene to have been identified, in SCA47 patients, is *PUM1,* encoding Pumilio1, a member of the PUMILIO/FBF RNA-binding protein family. The loss of *Pum1* led to an SCA1-like phenotype in mice by causing a 30–40% increase in wild-type Atxn1 protein levels in the cerebellum ^65^. These two proteins are functionally related: in SCA1 mice, the disease is more severe if one copy of *Pum1* is removed, whereas the motor phenotype of *Pum1-*heterozygous mice is improved by the removal of one copy of *Atxn1* ^65^. Mutations of *PUM1* were reported by Gennarino *et al*. in 15 patients, with different ages at disease onset (5 months to 50 years) and phenotypic presentations. A 50% loss of the protein resulted in a severe infantile disease and a developmental syndrome called Pumilio1-associated developmental disability, ataxia, and seizure (PADDAS), whereas the loss of 25% of the protein caused Pumilio1-related cerebellar ataxia (PRCA), with a later onset and incomplete penetrance ^66^.

Using whole-exome sequencing (WES), Nibbeling *et al*. identified the genes associated with SCA46 and SCA45: *PLD3* and *FAT2*, respectively. These genes, which are strongly expressed in the cerebellum, cause pure adult-onset cerebellar ataxia. However, in some cases, SCA46 patients may present with a sensory neuropathy ^67^.

Genes encoding ion channels are frequently involved in dominant cerebellar ataxia: mutations of *CACNA1A* are the most frequent genetic cause of autosomal dominant cerebellar ataxia in patients negative for polyQ SCAs, followed by other channel-coding genes, such as *KCND3*, *KCNC3*, and *KCNA1* ^68^. These genes predispose the patient to an earlier disease onset, intellectual deficiency, and slower disease progression ^68^. *CACNA1G* mutations, underlying SCA42 ^69^, lead to a slowly progressive pure ^70^ or complicated cerebellar ataxia associated with a spastic gait ^69^. Patients with earlier onset may also present facial dysmorphisms, microcephaly, digital abnormalities, and seizures ^71^.

Another channel gene implicated in SCA44 is *GRM1*, encoding the metabotropic glutamate receptor 1 (mGluR1) responsible for two phenotypic manifestations: adult-onset cerebellar ataxia in the presence of gain-of-function mutations and early onset ataxia with intellectual deficiency when associated with loss-of-function mutations ^72^.

The use of next-generation sequencing (NGS) techniques, such as WES, in everyday clinical practice has increased the diagnostic rate of complex neurological diseases ^73, 74^. The application of NGS in ataxia patients has revealed new SCA genes and improved our knowledge of the molecular pathways involved in cerebellar ataxia. It also affects the development of new therapeutic approaches: for example, elucidation of the role of mGluR1 in the excitability of Purkinje cells led to experiments modulating mGluR1 activity in SCA1 mice with drugs such as baclofen or the negative allosteric modulator JNJ16259685, both of which improved motor function ^75, 76^. As well, in a recent study by Bushart *et al*., the administration of potassium channel modulators induced improvement in SCA1 mice motor phenotype via electrophysiological changes ^77^; the authors also showed that chlorzoxazone and baclofen co-administration was well tolerated by SCA1 patients and should be considered as a promising approach for treating symptoms ^77^.

However, today, WES will not detect trinucleotide repeat disorders, mitochondrial DNA mutations, or large structural variants. Novel or *de novo* repeat expansions are still difficult to detect by short-read sequencing ^78^. Tandem repeats are usually discarded by NGS pipelines, but bioinformatics efforts will be made to detect them. Recently, a novel intronic expansion, a pentanucleotide-repeat in *SAMD12,* has been identified in familial myoclonic epilepsy ^78^. Long-read sequencing will probably allow one to identify more neurological repeat disorders.

## Therapeutic approaches in spinocerebellar ataxias

Over the last few years, several disease-modifying treatments have been tested with different outcomes. The most beneficial molecules to date are riluzole and valproic acid, whereas other drugs, such as lithium carbonate, trimethoprim-sulfamethoxazole, or zinc, have no significant effect ^79^. A randomized placebo-controlled clinical trial in 40 SCA patients (SCA1, 2, 6, 8, and 10) and 20 Friedreich ataxia patients treated with riluzole (100 mg/day) for 12 months revealed a one-point improvement in SARA score in treated patients compared to the placebo group ^80^. The use of valproic acid (1,200 mg/day) in SCA3 patients resulted in a SARA score at 12 weeks lower than that obtained for patients on placebo ^81^. However, further trials are required to identify the genotype associated with a positive response to riluzole (ClinicalTrials.gov Identifier: NCT03347344).

Based on the hypothesis that one of the main omega-3 polyunsaturated fatty acids of the cerebellum, docosahexaenoic acid (DHA), is present at low levels in SCA38 patients owing to *ELOVL5* mutations, a clinical trial of DHA (600 mg/day) was performed ^82^. This drug improved SARA score at 16 weeks in a small group of SCA38 patients and it also increased cerebellar metabolism, as shown by a brain 18-fluorodeoxyglucose positron emission tomography scan at 40 weeks ^82^.

However, the most encouraging and innovative strategies developed to date are RNA-targeting therapies for polyQ SCAs, which have been validated in several mouse models ^83, 84^. These approaches mostly use antisense oligonucleotides (ASOs) to downregulate levels of the pathological polyQ protein. SCA2 mice treated with intrathecal ASOs display improvements in motor abilities, a recovery of Purkinje cell firing frequency, and lower levels of mutated ATXN2 protein ^85^. ASOs have also been administered to SCA3 mice and were found to be well tolerated and to decrease the production of mutated protein ^86^. A correlation was also found with electrophysiological changes in Purkinje cells ^87^. These treatments may be used in patients with polyQ SCAs at early stages of the disease, even before the onset of symptoms. The long-term effects of decreasing levels of wild-type and mutated proteins and of decreasing the levels of other polyQ proteins remain unclear. The use of allele-specific ASOs may be required ^88^. A therapeutic approach based on ASOs is already at an advanced stage of development for Huntington disease ^89^, and a phase 1A clinical trial in patients has yielded promising results ^90^. ASO-based treatments are also being used for spinal muscular atrophy, in a different context, to retain exon 7 and produce a full-length SMN protein, with promising results ^91, 92^.

The advent of disease-modifying treatments for SCAs, including ASOs, will require the rapid identification and validation of robust biomarkers of disease progression or processes for the assessment of treatment response.

## Conclusion

This review summarizes recent clinical and genetic advances in dominant SCAs, including discoveries of novel SCAs, development of biomarkers, and therapeutic progress. The identification of biomarkers will be essential to demonstrate treatment efficacy. Future longitudinal studies based on multimodal approaches to elucidate the relationships between parameters will be required for the establishment of valid biomarkers. To date, gene suppression therapies are the most promising in polyQ SCAs, and clinical trials in the next few years are justified.

## Abbreviations

ASO, antisense oligonucleotide; CCAS, cerebellar cognitive affective/Schmahmann’s syndrome; CCFS, Composite Cerebellar Functional Severity; CSF, cerebrospinal fluid; DHA, docosahexaenoic acid; fMRI, functional MRI; GPx, glutathione peroxidase; mGluR1, metabotropic glutamate receptor 1; MRS, MRI spectroscopy; Nfl, neurofilament light; NGS, next-generation sequencing; polyQ, polyglutamine; SARA, Scale for the Assessment and Rating of Ataxia; SCA, spinocerebellar ataxia; WES, whole-exome sequencing.

## References

[1] Online Mendelian inheritance in man. OMIM Baltimore, MD: McKusick-Nathans Institute of Genetic Medicine, Johns Hopkins University. Reference Source

[2] Ruano L Melo C Silva MC: The global epidemiology of hereditary ataxia and spastic paraplegia: a systematic review of prevalence studies. *Neuroepidemiology.* 2014;42(3):174–83. 10.1159/000358801 24603320

[3] Durr A: Autosomal dominant cerebellar ataxias: polyglutamine expansions and beyond. *Lancet Neurol.* 2010;9(9):885–94. 10.1016/S1474-4422(10)70183-6 20723845

[4] MacDonald ME Ambrose CM Duyao MP A novel gene containing a trinucleotide repeat that is expanded and unstable on Huntington's disease chromosomes. The Huntington's Disease Collaborative Research Group. *Cell.* 1993;72(6):971–83. 10.1016/0092-8674(93)90585-E 8458085

[5] La Spada AR Wilson EM Lubahn DB: Androgen receptor gene mutations in X-linked spinal and bulbar muscular atrophy. *Nature.* 1991;352(6330):77–9. 10.1038/352077a0 2062380

[6] Stevanin G Dürr A Brice A: Clinical and molecular advances in autosomal dominant cerebellar ataxias: From genotype to phenotype and physiopathology. *Eur J Hum Genet.* 2000;8(1):4–18. 10.1038/sj.ejhg.5200403 10713882

[7] Orr HT Zoghbi HY: Trinucleotide repeat disorders. *Annu Rev Neurosci.* 2007;30:575–621. 10.1146/annurev.neuro.29.051605.113042 17417937

[8] Tezenas du Montcel S Durr A Bauer P: Modulation of the age at onset in spinocerebellar ataxia by CAG tracts in various genes. *Brain.* 2014;137(Pt 9):2444–55. 10.1093/brain/awu174 24972706 PMC4132646

[9] Tezenas du Montcel S Durr A Rakowicz M: Prediction of the age at onset in spinocerebellar ataxia type 1, 2, 3 and 6. *J Med Genet.* 2014;51(7):479–86. 10.1136/jmedgenet-2013-102200 24780882 PMC4078703

[10] Jacobi H Du Montcel ST Bauer P: Long-term disease progression in spinocerebellar ataxia types 1, 2, 3, and 6: a longitudinal cohort study. *Lancet Neurol.* 2015;14(11):1101–8. 10.1016/S1474-4422(15)00202-1 26377379

[11] Schmitz-Hübsch T du Montcel ST Baliko L: Scale for the assessment and rating of ataxia: development of a new clinical scale. *Neurology.* 2006;66(11):1717–20. 10.1212/01.wnl.0000219042.60538.92 16769946

[12] Schmitz-Hübsch T Fimmers R Rakowicz M: Responsiveness of different rating instruments in spinocerebellar ataxia patients. *Neurology.* 2010;74(8):678–84. 10.1212/WNL.0b013e3181d1a6c9 20177122

[13] Jacobi H Reetz K du Montcel ST: Biological and clinical characteristics of individuals at risk for spinocerebellar ataxia types 1, 2, 3, and 6 in the longitudinal RISCA study: analysis of baseline data. *Lancet Neurol.* 2013;12(7):650–8. 10.1016/S1474-4422(13)70104-2 23707147

[14] Monin ML Tezenas du Montcel S Marelli C: Survival and severity in dominant cerebellar ataxias. *Ann Clin Transl Neurol.* 2015;2(2):202–7. 10.1002/acn3.156 25750924 PMC4338960

[15] Ashizawa T Figueroa KP Perlman SL: Clinical characteristics of patients with spinocerebellar ataxias 1, 2, 3 and 6 in the US; a prospective observational study. *Orphanet J Rare Dis.* 2013;8:177. 10.1186/1750-1172-8-177 24225362 PMC3843578

[16] Orengo JP van der Heijden ME Hao S: Motor neuron degeneration correlates with respiratory dysfunction in SCA1. *Dis Model Mech.* 2018;11(2): pii: dmm032623. 10.1242/dmm.032623 29419414 PMC5894948

[17] Diallo A Jacobi H Cook A: Survival in patients with spinocerebellar ataxia types 1, 2, 3, and 6 (EUROSCA): a longitudinal cohort study. *Lancet Neurol.* 2018;17(4):327–34. 10.1016/S1474-4422(18)30042-5 29553382

[18] du Montcel ST Charles P Ribai P: Composite cerebellar functional severity score: validation of a quantitative score of cerebellar impairment. *Brain.* 2008;131(Pt 5):1352–61. 10.1093/brain/awn059 18378516

[19] Filipovic Pierucci A Mariotti C Panzeri M: Quantifiable evaluation of cerebellar signs in children. *Neurology.* 2015;84(12):1225–32. 10.1212/WNL.0000000000001403 25716360

[20] Tanguy Melac A Mariotti C Filipovic Pierucci A: Friedreich and dominant ataxias: quantitative differences in cerebellar dysfunction measurements. *J Neurol Neurosurg Psychiatry.* 2018;89(6):559–65. 10.1136/jnnp-2017-316964 29279305

[21] Reetz K Costa AS Mirzazade S: Genotype-specific patterns of atrophy progression are more sensitive than clinical decline in SCA1, SCA3 and SCA6. *Brain.* 2013;136(Pt 3):905–17. 10.1093/brain/aws369 23423669

[22] Mascalchi M Diciotti S Giannelli M: Progression of brain atrophy in spinocerebellar ataxia type 2: a longitudinal tensor-based morphometry study. *PLoS One.* 2014;9(2):e89410. 10.1371/journal.pone.0089410 24586758 PMC3934889

[23] Seidel K Siswanto S Brunt ERP: Brain pathology of spinocerebellar ataxias. *Acta Neuropathol.* 2012;124(1):1–21. 10.1007/s00401-012-1000-x 22684686

[24] Scherzed W Brunt ER Heinsen H: Pathoanatomy of cerebellar degeneration in spinocerebellar ataxia type 2 (SCA _2_) and type 3 (SCA _3_). *Cerebellum.* 2012;11(3):749–60. 10.1007/s12311-011-0340-8 22198871

[25] Reetz K Rodríguez-Labrada R Dogan I: Brain atrophy measures in preclinical and manifest spinocerebellar ataxia type 2. *Ann Clin Transl Neurol.* 2018;5(2):128–37. 10.1002/acn3.504 29468174 PMC5817824

[26] Hernandez-Castillo CR Galvez V Mercadillo R: Extensive White Matter Alterations and Its Correlations with Ataxia Severity in SCA 2 Patients. *PLoS One.* 2015;10(8):e0135449. 10.1371/journal.pone.0135449 26263162 PMC4532454

[27] Adanyeguh IM Perlbarg V Henry PG: Autosomal dominant cerebellar ataxias: Imaging biomarkers with high effect sizes. *Neuroimage Clin.* 2018;19:858–67. 10.1016/j.nicl.2018.06.011 29922574 PMC6005808

[28] Martins CR Jr Martinez ARM de Rezende TJR: Spinal Cord Damage in Spinocerebellar Ataxia Type 1. *Cerebellum.* 2017;16(4):792–6. 10.1007/s12311-017-0854-9 28386793

[29] Pedroso JL de Souza PV Pinto WB: SCA1 patients may present as hereditary spastic paraplegia and must be included in spastic-ataxias group. *Parkinsonism Relat Disord.* 2015;21(10):1243–6. 10.1016/j.parkreldis.2015.07.015 26231471

[30] Fahl CN Branco LM Bergo FP: Spinal cord damage in Machado-Joseph disease. *Cerebellum.* 2015;14(2):128–32. 10.1007/s12311-014-0619-7 25370748

[31] Joers JM Deelchand DK Lyu T: Neurochemical abnormalities in premanifest and early spinocerebellar ataxias. *Ann Neurol.* 2018;83(4):816–29. 10.1002/ana.25212 29575033 PMC5912959

[32] Adanyeguh IM Henry PG Nguyen TM: *In vivo* neurometabolic profiling in patients with spinocerebellar ataxia types 1, 2, 3, and 7. *Mov Disord.* 2015;30(5):662–70. 10.1002/mds.26181 25773989 PMC4397159

[33] Cocozza S Saccà F Cervo A: Modifications of resting state networks in spinocerebellar ataxia type 2. *Mov Disord.* 2015;30(10):1382–90. 10.1002/mds.26284 26094751

[34] Guell X Gabrieli JDE Schmahmann JD: Triple representation of language, working memory, social and emotion processing in the cerebellum: convergent evidence from task and seed-based resting-state fMRI analyses in a single large cohort. *Neuroimage.* 2018;172:437–49. 10.1016/j.neuroimage.2018.01.082 29408539 PMC5910233

[35] Stefanescu MR Dohnalek M Maderwald S: Structural and functional MRI abnormalities of cerebellar cortex and nuclei in SCA3, SCA6 and Friedreich's ataxia. *Brain.* 2015;138(Pt 5):1182–97. 10.1093/brain/awv064 25818870 PMC5963415

[36] Deistung A Stefanescu MR Ernst TM: Structural and Functional Magnetic Resonance Imaging of the Cerebellum: Considerations for Assessing Cerebellar Ataxias. *Cerebellum.* 2016;15(1):21–5. 10.1007/s12311-015-0738-9 26521073

[37] Duarte JV Faustino R Lobo M: Parametric fMRI of paced motor responses uncovers novel whole-brain imaging biomarkers in spinocerebellar ataxia type 3. *Hum Brain Mapp.* 2016;37(10):3656–68. 10.1002/hbm.23266 27273236 PMC6867469

[38] Wu C Chen DB Feng L: Oculomotor deficits in spinocerebellar ataxia type 3: Potential biomarkers of preclinical detection and disease progression. *CNS Neurosci Ther.* 2017;23(4):321–8. 10.1111/cns.12676 28195427 PMC6492748

[39] Raposo M Vasconcelos J Bettencourt C: Nystagmus as an early ocular alteration in Machado-Joseph disease (MJD/SCA3). *BMC Neurol.* 2014;14:17. 10.1186/1471-2377-14-17 24450306 PMC3901765

[40] Moscovich M Okun MS Favilla C: Clinical evaluation of eye movements in spinocerebellar ataxias: a prospective multicenter study. *J Neuroophthalmol.* 2015;35(1):16–21. 10.1097/WNO.0000000000000167 25259863 PMC4675453

[41] Velázquez-Pérez L Rodríguez-Labrada R Cruz-Rivas EM: Comprehensive study of early features in spinocerebellar ataxia 2: Delineating the prodromal stage of the disease. *Cerebellum.* 2014;13(5):568–79. 10.1007/s12311-014-0574-3 24906824

[42] Rodríguez-Labrada R Velázquez-Pérez L Auburger G: Spinocerebellar ataxia type 2: Measures of saccade changes improve power for clinical trials. *Mov Disord.* 2016;31(4):570–8. 10.1002/mds.26532 26846400

[43] Cunha-Santos J Duarte-Neves J Carmona V: Caloric restriction blocks neuropathology and motor deficits in Machado-Joseph disease mouse models through SIRT1 pathway. *Nat Commun.* 2016;7:11445. 10.1038/ncomms11445 27165717 PMC4865854

[44] Wu YL Chang JC Lin WY: Caffeic acid and resveratrol ameliorate cellular damage in cell and Drosophila models of spinocerebellar ataxia type 3 through upregulation of Nrf2 pathway. *Free Radic Biol Med.* 2018;115:309–17. 10.1016/j.freeradbiomed.2017.12.011 29247688

[45] Ghorbani Z Farahani RM Aliaghaei A: Resveratrol Protects Purkinje Neurons and Restores Muscle Activity in Rat Model of Cerebellar Ataxia. *J Mol Neurosci.* 2018;65(1):35–42. 10.1007/s12031-018-1065-7 29713949

[46] Kazachkova N Raposo M Ramos A: Promoter Variant Alters Expression of the Autophagic *BECN _1_* Gene: Implications for Clinical Manifestations of Machado-Joseph Disease. *Cerebellum.* 2017;16(5–6):957–63. 10.1007/s12311-017-0875-4 28699106

[47] Raposo M Ramos A Santos C: Accumulation of Mitochondrial DNA Common Deletion Since The Preataxic Stage of Machado-Joseph Disease. *Mol Neurobiol.* 2018;1–6. 10.1007/s12035-018-1069-x 29679261

[48] Pacheco LS da Silveira AF Trott A: Association between Machado-Joseph disease and oxidative stress biomarkers. *Mutat Res.* 2013;757(2):99–103. 10.1016/j.mrgentox.2013.06.023 23994570

[49] de Assis AM Saute JAM Longoni A: Peripheral Oxidative Stress Biomarkers in Spinocerebellar Ataxia Type 3/Machado-Joseph Disease. *Front Neurol.* 2017;8:485. 10.3389/fneur.2017.00485 28979235 PMC5611390

[50] Evert BO Schelhaas J Fleischer H: Neuronal intranuclear inclusions, dysregulation of cytokine expression and cell death in spinocerebellar ataxia type 3. *Clin Neuropathol.* 2006;25(6):272–81. 17140157

[51] da Silva Carvalho G Saute JAM Haas CB: Cytokines in Machado Joseph Disease/Spinocerebellar Ataxia 3. *Cerebellum.* 2016;15(4):518–25. 10.1007/s12311-015-0719-z 26395908

[52] Raposo M Bettencourt C Ramos A: Promoter Variation and Expression Levels of Inflammatory Genes *IL1A*, *IL1B*, *IL6* and *TNF* in Blood of Spinocerebellar Ataxia Type 3 (SCA _3_) Patients. *Neuromolecular Med.* 2017;19(1):41–5. 10.1007/s12017-016-8416-8 27246313

[53] Zhang H Wu LM Wu J: Cross-talk between apolipoprotein E and cytokines. *Mediators Inflamm.* 2011;2011:949072. 10.1155/2011/949072 21772670 PMC3136159

[54] Wilke C Bender F Hayer SN: Serum neurofilament light is increased in multiple system atrophy of cerebellar type and in repeat-expansion spinocerebellar ataxias: a pilot study. *J Neurol.* 2018;265(7):1618–24. 10.1007/s00415-018-8893-9 29737427

[55] Menke RA Gray E Lu CH: CSF neurofilament light chain reflects corticospinal tract degeneration in ALS. *Ann Clin Transl Neurol.* 2015;2(7):748–55. 10.1002/acn3.212 26273687 PMC4531057

[56] Byrne LM Rodrigues FB Blennow K: Neurofilament light protein in blood as a potential biomarker of neurodegeneration in Huntington's disease: a retrospective cohort analysis. *Lancet Neurol.* 2017;16(8):601–9. 10.1016/S1474-4422(17)30124-2 28601473 PMC5507767

[57] Rossi D Volanti P Brambilla L: CSF neurofilament proteins as diagnostic and prognostic biomarkers for amyotrophic lateral sclerosis. *J Neurol.* 2018;265(3):510–21. 10.1007/s00415-017-8730-6 29322259

[58] Brouillette AM Öz G Gomez CM: Cerebrospinal Fluid Biomarkers in Spinocerebellar Ataxia: A Pilot Study. *Dis Markers.* 2015;2015:413098. 10.1155/2015/413098 26265793 PMC4525756

[59] Bürk K Globas C Bösch S: Cognitive deficits in spinocerebellar ataxia type 1, 2, and 3. *J Neurol.* 2003;250(2):207–11. 10.1007/s00415-003-0976-5 12574952

[60] Moriarty A Cook A Hunt H: A longitudinal investigation into cognition and disease progression in spinocerebellar ataxia types 1, 2, 3, 6, and 7. *Orphanet J Rare Dis.* 2016;11(1):82. 10.1186/s13023-016-0447-6 27333979 PMC4917932

[61] Jacobi H du Montcel ST Bauer P: Long-term evolution of patient-reported outcome measures in spinocerebellar ataxias. *J Neurol.* 2018;265(9):2040–51. 10.1007/s00415-018-8954-0 29959555

[62] Fancellu R Paridi D Tomasello C: Longitudinal study of cognitive and psychiatric functions in spinocerebellar ataxia types 1 and 2. *J Neurol.* 2013;260(12):3134–43. 10.1007/s00415-013-7138-1 24122064

[63] Pereira L Airan RD Fishman A: Resting-state functional connectivity and cognitive dysfunction correlations in spinocerebelellar ataxia type 6 (SCA6). *Hum Brain Mapp.* 2017;38(6):3001–10. 10.1002/hbm.23568 28295805 PMC6866919

[64] Hoche F Guell X Vangel MG: The cerebellar cognitive affective/Schmahmann syndrome scale. *Brain.* 2018;141(1):248–70. 10.1093/brain/awx317 29206893 PMC5837248

[65] Gennarino VA Singh RK White JJ: *Pumilio1* haploinsufficiency leads to SCA1-like neurodegeneration by increasing wild-type Ataxin1 levels. *Cell.* 2015;160(6):1087–98. 10.1016/j.cell.2015.02.012 25768905 PMC4383046

[66] Gennarino VA Palmer EE McDonell LM: A Mild *PUM1* Mutation Is Associated with Adult-Onset Ataxia, whereas Haploinsufficiency Causes Developmental Delay and Seizures. *Cell.* 2018;172(5):924–936.e11. 10.1016/j.cell.2018.02.006 29474920 PMC5832058

[67] Nibbeling EAR Duarri A Verschuuren-Bemelmans CC: Exome sequencing and network analysis identifies shared mechanisms underlying spinocerebellar ataxia. *Brain.* 2017;140(11):2860–78. 10.1093/brain/awx251 29053796

[68] Coutelier M Coarelli G Monin ML: A panel study on patients with dominant cerebellar ataxia highlights the frequency of channelopathies. *Brain.* 2017;140(6):1579–94. 10.1093/brain/awx081 28444220

[69] Coutelier M Blesneac I Monteil A: A Recurrent Mutation in *CACNA1G* Alters Cav3.1 T-Type Calcium-Channel Conduction and Causes Autosomal-Dominant Cerebellar Ataxia. *Am J Hum Genet.* 2015;97(5):726–37. 10.1016/j.ajhg.2015.09.007 26456284 PMC4667105

[70] Kimura M Yabe I Hama Y: SCA42 mutation analysis in a case series of Japanese patients with spinocerebellar ataxia. *J Hum Genet.* 2017;62(9):857–9. 10.1038/jhg.2017.51 28490766

[71] Chemin J Siquier-Pernet K Nicouleau M: *De novo* mutation screening in childhood-onset cerebellar atrophy identifies gain-of-function mutations in the *CACNA1G* calcium channel gene. *Brain.* 2018;141(7):1998–2013. 10.1093/brain/awy145 29878067

[72] Watson LM Bamber E Schnekenberg RP: Dominant Mutations in *GRM1* Cause Spinocerebellar Ataxia Type 44. *Am J Hum Genet.* 2017;101(3):451–458. 10.1016/j.ajhg.2017.08.005 28886343 PMC5591020

[73] Jiang T Tan MS Tan L: Application of next-generation sequencing technologies in Neurology. *Ann Transl Med.* 2014;2(12):125. 10.3978/j.issn.2305-5839.2014.11.11 25568878 PMC4260045

[74] Lee H Deignan JL Dorrani N: Clinical exome sequencing for genetic identification of rare Mendelian disorders. *JAMA.* 2014;312(18):1880–7. 10.1001/jama.2014.14604 25326637 PMC4278636

[75] Shuvaev AN Hosoi N Sato Y: Progressive impairment of cerebellar mGluR signalling and its therapeutic potential for cerebellar ataxia in spinocerebellar ataxia type 1 model mice. *J Physiol.* 2017;595(1):141–64. 10.1113/JP272950 27440721 PMC5199750

[76] Power EM Morales A Empson RM: Prolonged Type 1 Metabotropic Glutamate Receptor Dependent Synaptic Signaling Contributes to Spino-Cerebellar Ataxia Type 1. *J Neurosci.* 2016;36(18):4910–6. 10.1523/JNEUROSCI.3953-15.2016 27147646 PMC6601852

[77] Bushart DD Chopra R Singh V: Targeting potassium channels to treat cerebellar ataxia. *Ann Clin Transl Neurol.* 2018;5(3):297–314. 10.1002/acn3.527 29560375 PMC5846455

[78] Ishiura H Doi K Mitsui J: Expansions of intronic TTTCA and TTTTA repeats in benign adult familial myoclonic epilepsy. *Nat Genet.* 2018;50(4):581–90. 10.1038/s41588-018-0067-2 29507423

[79] Zesiewicz TA Wilmot G Kuo SH: Comprehensive systematic review summary: Treatment of cerebellar motor dysfunction and ataxia: Report of the Guideline Development, Dissemination, and Implementation Subcommittee of the American Academy of Neurology. *Neurology.* 2018;90(10):464–71. 10.1212/WNL.0000000000005055 29440566 PMC5863491

[80] Romano S Coarelli G Marcotulli C: Riluzole in patients with hereditary cerebellar ataxia: a randomised, double-blind, placebo-controlled trial. *Lancet Neurol.* 2015;14(10):985–91. 10.1016/S1474-4422(15)00201-X 26321318

[81] Lei LF Yang GP Wang JL: Safety and efficacy of valproic acid treatment in SCA _3_/MJD patients. *Parkinsonism Relat Disord.* 2016;26:55–61. 10.1016/j.parkreldis.2016.03.005 26997655

[82] Manes M Alberici A Di Gregorio E: Docosahexaenoic acid is a beneficial replacement treatment for spinocerebellar ataxia 38. *Ann Neurol.* 2017;82(4):615–21. 10.1002/ana.25059 28976605 PMC5698802

[83] Keiser MS Kordasiewicz HB McBride JL: Gene suppression strategies for dominantly inherited neurodegenerative diseases: lessons from Huntington's disease and spinocerebellar ataxia. *Hum Mol Genet.* 2016;25(R1):R53–64. 10.1093/hmg/ddv442 26503961 PMC4802374

[84] Matos CA de Almeida LP Nóbrega C: Machado-Joseph disease/spinocerebellar ataxia type 3: lessons from disease pathogenesis and clues into therapy. *J Neurochem.* 2018. 10.1111/jnc.14541 29959858

[85] Scoles DR Meera P Schneider MD: Antisense oligonucleotide therapy for spinocerebellar ataxia type 2. *Nature.* 2017;544(7650):362–6. 10.1038/nature22044 28405024 PMC6625650

[86] Moore LR Rajpal G Dillingham IT: Evaluation of Antisense Oligonucleotides Targeting ATXN3 in SCA3 Mouse Models. *Mol Ther Nucleic Acids.* 2017;7:200–10. 10.1016/j.omtn.2017.04.005 28624196 PMC5415970

[87] McLoughlin HS Moore LR Chopra R: Oligonucleotide therapy mitigates disease in spinocerebellar ataxia type 3 mice. *Ann Neurol.* 2018;84(1):64–77. 10.1002/ana.25264 29908063 PMC6119475

[88] Toonen LJA Rigo F van Attikum H: Antisense Oligonucleotide-Mediated Removal of the Polyglutamine Repeat in Spinocerebellar Ataxia Type 3 Mice. *Mol Ther Nucleic Acids.* 2017;8:232–42. 10.1016/j.omtn.2017.06.019 28918024 PMC5504086

[89] Skotte NH Southwell AL Østergaard ME: Allele-specific suppression of mutant huntingtin using antisense oligonucleotides: providing a therapeutic option for all Huntington disease patients. *PLoS One.* 2014;9(9):e107434. 10.1371/journal.pone.0107434 25207939 PMC4160241

[90] Wild EJ Tabrizi SJ: Therapies targeting DNA and RNA in Huntington's disease. *Lancet Neurol.* 2017;16(10):837–47. 10.1016/S1474-4422(17)30280-6 28920889 PMC5604739

[91] Finkel RS Mercuri E Darras BT: Nusinersen versus Sham Control in Infantile-Onset Spinal Muscular Atrophy. *N Engl J Med.* 2017;377(18):1723–32. 10.1056/NEJMoa1702752 29091570

[92] Mercuri E Finkel R Kirschner J: Efficacy and safety of nusinersen in children with later-onset spinal muscular atrophy (SMA): End of study results from the phase 3 CHERISH study. *Neuromuscular Disorders.* 2017;27(2):S210 10.1016/j.nmd.2017.06.418

